# The Mechanism of Chlorantraniliprole Resistance and Detoxification in *Trichogramma chilonis* (Hymenoptera: Trichogrammatidae)

**DOI:** 10.1093/jisesa/ieac044

**Published:** 2022-07-29

**Authors:** Wenya Zhu, Juan Wang, Ye Zhang

**Affiliations:** College of Plant Protection, Shanxi Agricultural University, Taiyuan 030031, China; College of Plant Protection, Shanxi Agricultural University, Taiyuan 030031, China; College of Plant Protection, Shanxi Agricultural University, Taiyuan 030031, China

**Keywords:** chlorantraniliprole, cytochrome P450, glutathione, control, detoxification, resistance, genes

## Abstract

Parasitic *Trichogramma chilonis* Ishii, an egg parasitoid of *Grapholita molesta*, is a critical agent for biological control of insect pests in crop plants. However, the efficiency of *T. chilonis* is influenced by its resistance to the common pesticide chlorantraniliprole. To elucidate the chlorantraniliprole detoxification mechanism, differentially expressed genes (DEGs) related to chlorantraniliprole resistance were studied at different developmental stages of the wasp. Individuals of *T. chilonis* were grouped and treated with chlorantraniliprole at different developmental stages. Untreated wasps were used as controls. Transcriptomic analysis identified the DEGs associated with chlorantraniliprole resistance and detoxification in *T. chilonis*. A total of 1,483 DEGs were associated with chlorantraniliprole resistance at all developmental stages. DEGs that correlated with chlorantraniliprole sensitivity of *T. chilonis* at different developmental stages were distinct and had various functions. The newly identified DEGs are involved in cytochrome P450- and glutathione metabolism-related pathways, which were predicted to contribute to chlorantraniliprole detoxification. Chlorantraniliprole detoxification by *T. chilonis* was associated with cytochrome P450- and glutathione-related pathways. Our findings may be useful for balancing chemical and biological control practices aimed to optimize agricultural production.

Biological control has become an important element of pest management systems to meet the increasing demand for more sustainable pest-control methods (Lacey et al. 2015, Abram and Moffat 2018). *Trichogramma chilonis* Ishii is a valuable natural insect resource that has been widely used for the biological management of various agricultural and forestry pests, such as *Helicoverpa armigera* (Hübner), *Corcyra cephalonica* (Stainton), and *Chilo suppressalis* (Walker) (Liu et al. 2016, Yang et al. 2016, Tang et al. 2017, Zang et al. 2021). Further, numerous studies have focused on *T. chilonis* owing to its wide application range and area of distribution, as well as its broad effectiveness against various insect pests.

Chlorantraniliprole, a key anthranilic diamide, is a novel chemical insecticide that is reportedly the most active compound for controlling lepidopteran pests (Carscallen et al. 2019, He et al. 2019). Chlorantraniliprole can induce feeding cessation and muscle paralysis, resulting in death by binding with ryanodine and promoting calcium release (Plata-Rueda et al. 2019). Although there is no cross-resistance between chlorantraniliprole and other insecticides, it is still necessary to investigate the detoxification and resistance mechanisms at play in different types of insects.

Previous studies have focused on chlorantraniliprole resistance-related genes in pests. For example, Lin et al. (2013) performed transcriptome analysis on the diamondback moth (*Plutella xylostella*) and provided valuable resources for pest control by identifying several genes specifically related to chlorantraniliprole resistance. However, although chemical toxicity to beneficial arthropods has improved over the past decades, there is insufficient data on the toxic effects of pesticides on *T. chilonis* (Wang et al. 2012a, Ko et al. 2015, Wang et al. 2016). Furthermore, chlorantraniliprole detoxification in beneficial insects is of great importance for the development of effective and efficient biological control. Filtering the resistance of *T. chilonis* to insecticides might minimize the adverse effects of pesticides on *T. chilonis*, thereby improving the efficiency of biological control management. The identification of hub genes correlated with detoxification and resistance to pesticides would be helpful for understanding the underlying mechanisms and filtering resistant species.

The sensitivity of *T. chilonis* to pesticides depends not only on the chemical structure and physicochemical properties of pesticides but is also associated with the developmental stage and biological characteristics of *T. chilonis* (Casida and Durkin 2017, Seesen et al. 2020, Wang et al. 2020). The objective of this study was to identify chlorantraniliprole detoxification-related genes and pathways in *T. chilonis* at different developmental stages, thereby providing a basis for the potential co-application of chlorantraniliprole and *T. chilonis*.

## Materials and Methods

### Sample Resources and Treatment

Eggs of the *Grapholita molesta* egg-parasitoid *T. chilonis* were collected from a pear orchard in Hongzhiyi Town, Yanhu District, Yuncheng City, Shanxi Province, China. After hatching, *T. chilonis* adults were maintained at 25 ± 1°C, and 75 ± 5% relative humidity, under a 15:9 h (light:dark) photoperiod.

Eggs of *Corcyra cephalonica* were used as hosts for breeding. The *C. cephalonica* population was kept in the laboratory for a long time without exposure to any pesticides. Briefly, fresh and clean *C. cephalonica* eggs were irradiated under ultraviolet lamp for 30 min to kill the embryos in the eggs. Then, the inactivated *C. cephalonica* eggs were evenly scattered on a card paper coated with white latex (4.0 cm × 2.5 cm) without stacking or overlapping among them, and the non-parasitic egg cards were formed after drying. Adult insects were excluded after 4 h of seeding at a ratio of 1:20 (insects:eggs), and the seeded insects were maintained at 25 ± 1°C, 75 ± 5% relative humidity, under a 15:9 h (light: dark) photoperiod for 1, 4, and 7 d. *T. chilonis* specimens at different developmental stages (i.e., 1, 4, and 7 d) were soaked in chlorantraniliprole for 5 s. The chlorantraniliprole dose was selected based on the LD_50_ value. The control group was treated with acetone. The insects in each group were frozen in liquid nitrogen and stored at −80°C for subsequent RNA extraction. The *T. chilonis* strains retained are listed in Table 1.

**Table 1.** Specific treatment of different groups

Groups	Treatments
CK	Control
LV	Live insects after 4 h of chlorantraniliprole treatment
T2	The first day after the treatment of chlorantraniliprole
T3	The fourth day after the treatment of chlorantraniliprole
T4	The seventh day after the treatment of chlorantraniliprole
T5	The first day of the common development
T6	The fourth day of the common development
T7	The seventh day of the common development

For the determination of LD_50_, chlorantrantranamide toxicity to *T. chilonis* adult wasps was determined by the drug-membrane method. The pesticide concentration range was determined by a preliminary test; then, each dose was diluted into five concentrations gradients with acetone, and acetone was used as control. Drugs (0.5 ml) were added into a tube (cross-sectional area of 54.6 cm^2^) and rotated at a constant speed to ensure an even distribution to construct the drug membrane. After acetone was volatilized, adult wasps (100 ± 10) were seeded into each tube 6 h after emergence, and fed with 10% honey water. The tubes were sealed with black cloth and maintained at 25 ± 1°C, 75 ± 5% relative humidity, under a 15:9 h (light: dark) photoperiod. After 8 h, the number of alive and dead adult insects were counted and the LD_50_ was calculated. All treatments were performed in triplicate.

### RNA Isolation, Library Preparation, and Sequencing

Total RNA was extracted using TRIzol Reagent (B511311; Sangon; Shanghai, China). The RNA concentration was assessed using a Qubit 2.0 RNA detection kit (Q32855; Life Technologies, USA) and RNA purity and integrity were assessed by agarose gel electrophoresis. The cDNA libraries were constructed using the NEBNext Ultra RNA Library Prep Kit for Illumina (Illumina, USA) and then amplified by PCR. Sequencing was performed on the Illumina NovaSeq6000 platform (Illumina, USA).

### Data Assessment and Quality Control

Raw sequencing data contained low quality sequences with adapter that were assessed using FastQC (version 0.11.2) and cleaned with Trimmomatic (version 0.36) (Bolger et al. 2014) as follows: 1) Removing sequences with N bases; 2) Removing the adapter sequence in reads; 3) Removal of low quality bases from 3ʹ to 5ʹ of reads (Q value < 20); 4) Removal of low quality bases from 5ʹ to 3ʹ of reads (Q value < 20); 5) Removal of bases with read-tail mass value greater than 20 by the sliding window method (window size was 5 bp); 6) Removing reads less than 35 nt in length and their paired reads.

Ten thousand sequences were randomly selected from the clean data obtained for BLASTN with the National Center for Biotechnology Information (NCBI) Nucleotide Sequence Database. BLASTN results with e ≤ 1e−10, similarity > 90%, and coverage > 80% were used for calculating species distribution and pollution detection.

### Sequence Assembly and Comparison

Clean data were assembled de novo using Trinity (version 2.4.0). The parameter ‘min_kmer_cov 2’ was used to eliminate single-occurrence k-mers heavily enriched in sequencing errors (Haas et al. 2013). The other parameters were set at the corresponding default values. Transcripts assembled by Trinity were subjected to redundancy removal, and the longest transcript in each transcript cluster was selected as the unigene used as reference sequence. The quality control sequence and the reference sequence were compared using Bowtie2 (version 2.3.2) (Langmead and Salzberg 2012) and the results were analyzed with RseQC (version 2.6.1), as previously reported (Wang et al. 2012b).

### Evaluation of Gene Expression Level

The transcript per million (TPM) value was used to estimate the percentage of transcripts in the RNA pool, considering the effects of sequence depth, gene length, and samples on read counting. The TPM value was calculated using the following equation:


$$\begin{array}{l} TPM_{i}=\frac{X_{i}}{L_{i}}*\frac{1}{\sum_{j}\frac{X_{j}}{L_{j}}}*10^{6} \end{array}$$

$X_{i}$ =*total exon fragments/reads*$L_{i}=\frac{exon\;length}{KB}$

### Principal Component Analysis

Principal component analysis (PCA) was performed to reduce the dimensionality and maintain the features of the data. PCA was conducted using the ‘vegan’ package in the R software (https://mirrors.tuna.tsinghua.edu.cn/CRAN/web/packages/vegan/index.html, version 2.0-10).

### Differentially Expressed Genes and Function Enrichment

Differentially expressed genes (DEGs) were obtained by comparing the genes among different groups using DEGseq (http://master.bioconductor.org/packages/release/bioc/html/DEGseq.html, version 1.26.0) with a significance level of less than 0.05, and |Fold Change| > 2 (Wang et al. 2010). Functional enrichment of DEGs was conducted using clusterProfiler (http://master.bioconductor.org/packages/release/bioc/html/clusterProfiler.html, version 3.0.5) (Wu et al. 2021). Q values < 0.05 indicated significant enrichment.

### Data Access

The RNA-seq raw data were submitted to the NCBI Sequence Read Archive (SRA) under accession number PRJNA838766.

### qRT-PCR Assay

The expression level of the key genes associated with cytochrome P450 and carboxylesterase metabolism were validated by qRT-PCR. The genes included TRINITY_DN48242_c7_g4 (up-regulated in LV vs. CK), TRINITY_DN45356_c0_g2 (up-regulated in LV vs. CK), and TRINITY_DN31375_c0_g1 (up-regulated in T5 vs. T2). Total RNA was extracted using the TRIzol reagent (Tiangen, China). One microgram of the total RNA was used for reverse transcription. The qPCR reaction system consisted of 5 μl of 2 × SG Green qPCR Mix, 0.5 μl of each primer, l μl of cDNA, and water (nuclease-free) prepared up to 10 μl. Primer sequences used are listed in Table 2. The PCR reaction program consisted of one cycle of initial denaturation at 95°C for 3 min, 40 cycles of denaturation at 95°C for 10 s, and annealing and extension at 60°C for 30 s. The relative expression level of genes were calculated using the 2^-ΔΔCt^ method.

**Table 2.** List of primers used for real-time PCR

Primers names	Upstream base sequence	Downstream base sequence
rps23	TGCCATCCGAAAGTGTGTCA	TACGACCGAATCCTGCAACC
TRINITY_DN48242_c7_g4	CCTCTGAAACACCCACCTAAG	CCATGAAACAATTCGCTCC
TRINITY_DN31375_c0_g1	GCTCGGCTACTGGGACATT	ATGAGGTAGGGCAGGTTGG
TRINITY_DN45356_c0_g2	GCAAAGCGACAAAGGGAG	CAAACGAGAACGGCGATAA

## Results

### Quality Control and Read Assembly

RNA sequencing generated a total of 44,771,700, 48,362,596, 42,595,522, 49,601,330, 43,741,914, 37,399,410, 41,099,796, and 45,797,862 raw reads for the CK, T6, T7, T4, T5, T2, T3, and LV groups, respectively (Table 3). To ensure the quality of the data analysis, raw data were filtered to obtain clean data. The results of quality control are shown in Table 3. The total clean reads in the CK, T6, T7, T4, T5, T2, T3, and LV groups were 42,647,442 (average length 141.86 bp), 45,761,140 (average length 141.28 bp), 40,370,436 (average length 140.95 bp), 467,34,344 (average length 140.65 bp), 41,829,994 (average length 141.40 bp), 35,460,450 (average length 142.24 bp), 39,328,112 (average length 142.84 bp), and 43,960,060 (average length 143.22 bp), respectively.

**Table 3.** Statistical results of data after quality control

	CK	T6	T7	T4	T5	T2	T3	LV
Total Raw Reads Count (#)	44771700	48362596	42595522	49601330	43741914	37399410	41099796	45797862
Total Clean Reads Count (#)	42647442	45761140	40370436	46734344	41829994	35460450	39328112	43960060
Total Raw Bases Count (bp)	6715755000	7254389400	6389328300	7440199500	6561287100	5609911500	6164969400	6869679300
Total Clean Bases Count (bp)	6049760673	6465224758	5690341509	6573241541	5914834206	5043739661	5617703642	6295881639
Average Raw Read Length (bp)	150	150	150	150	150	150	150	150
Average Clean Read Length (bp)	141.86	141.28	140.95	140.65	141.4	142.24	142.84	143.22
Q10 Bases Count (bp)	6016492454	6.42E+09	5.66E+09	6.57E+09	5.88E+09	5.04E+09	5.62E+09	6.3E+09
Q10 Bases Ratio (%)	99.45%	99.37%	99.41%	100.00%	99.47%	100.00%	100.00%	100.00%
Q20 Bases Count (bp)	5902797845	6.28E+09	5.54E+09	6.45E+09	5.78E+09	4.96E+09	5.52E+09	6.18E+09
Q20 Bases Ratio (%)	97.57%	97.21%	97.41%	98.10%	97.68%	98.28%	98.32%	98.17%
Q30 Bases Count (bp)	5481813394	5.79E+09	5.13E+09	6.09E+09	5.38E+09	4.7E+09	5.25E+09	5.87E+09
Q30 Bases Ratio (%)	90.61%	89.56%	90.18%	92.66%	91.00%	93.17%	93.48%	93.20%
N Bases Count (bp)	2031379	2088653	1924929	29203	2049893	22459	23318	22521
N Bases Ratio (%)	0.03%	0.03%	0.03%	0.00%	0.03%	0.00%	0.00%	0.00%
GC Bases Count (bp)	2512022539	2.67E+09	2.38E+09	3.06E+09	2.53E+09	2.39E+09	2.68E+09	2.89E+09
GC Bases Ratio (%)	41.52%	41.35%	41.78%	46.57%	42.78%	47.43%	47.75%	45.87%

Q{N0} Base Count: The number of bases with the quality over {N0}; Q{N0} Base Ratio: The percentage of bases with the quality over {N0}.

The de novo Trinity assembly yielded 274,964 transcripts and 148,866 unigenes that were used as reference sequences in the following analyses. The average length of the obtained unigenes was 749.42 bp with a maximum and minimum lengths of 22,698 and 201 bp, respectively (Table 4). N50 and N90 were used to evaluate the splicing effect. N50: the spliced transcripts were sorted from longest to shortest, and the length of the transcripts was accumulated until the length of the spliced transcripts was not less than 50% of the total length of N50. N90: the spliced transcripts were sorted from longest to shortest, and the length of the transcripts was accumulated until the length of the spliced transcripts was not less than 90% of the total length of N90.

**Table 4.** Statistical results of assembly

	No.	>=500bp	>=1000bp	N50	N90	Max length	Min length	Total length	Average length
Transcript	274964	132811	84223	2351	377	22698	201	304356937	1106.9
Unigene	148866	51884	26903	1423	268	22698	201	111562727	749.42
Transcript	274964	132811	84223	2351	377	22698	201	304356937	1106.9
Unigene	148866	51884	26903	1423	268	22698	201	111562727	749.42

Transcript: the spliced transcript sequences; Unigene: the spliced transcript sequence with redundancy removed. N50: the spliced transcripts were sorted from longest to shortest length, and the length of the transcripts was accumulated until the length of the spliced transcripts was not less than 50% of the total length of N50. N90: the spliced transcripts were sorted from longest to shortest length, and the length of the transcripts was accumulated until the length of the spliced transcripts was not less than 90% of the total length of N90.

### DEGs in the Groups

**Fig. 1.**
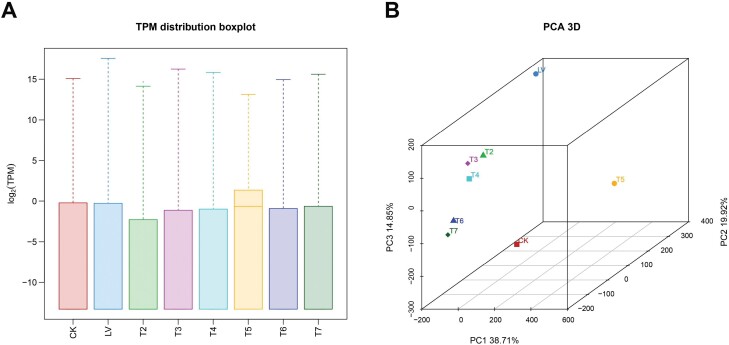
DEGs between different paired groups. (A) Gene expression in different groups. Different colors represent samples from different groups. (B) PCA conducted to evaluate the differences between different groups. The color and sharpness of the points distinguish the groups.

The gene expression levels of different groups are summarized in Fig. 1A, and PCA was performed to evaluate the differences among groups. The samples with and without chlorantraniliprole treatment differed remarkably, and in the absence of chlorantraniliprole, group T5 was dispersed from groups T6 and T7 (Fig. 1B).

**Fig. 2.**
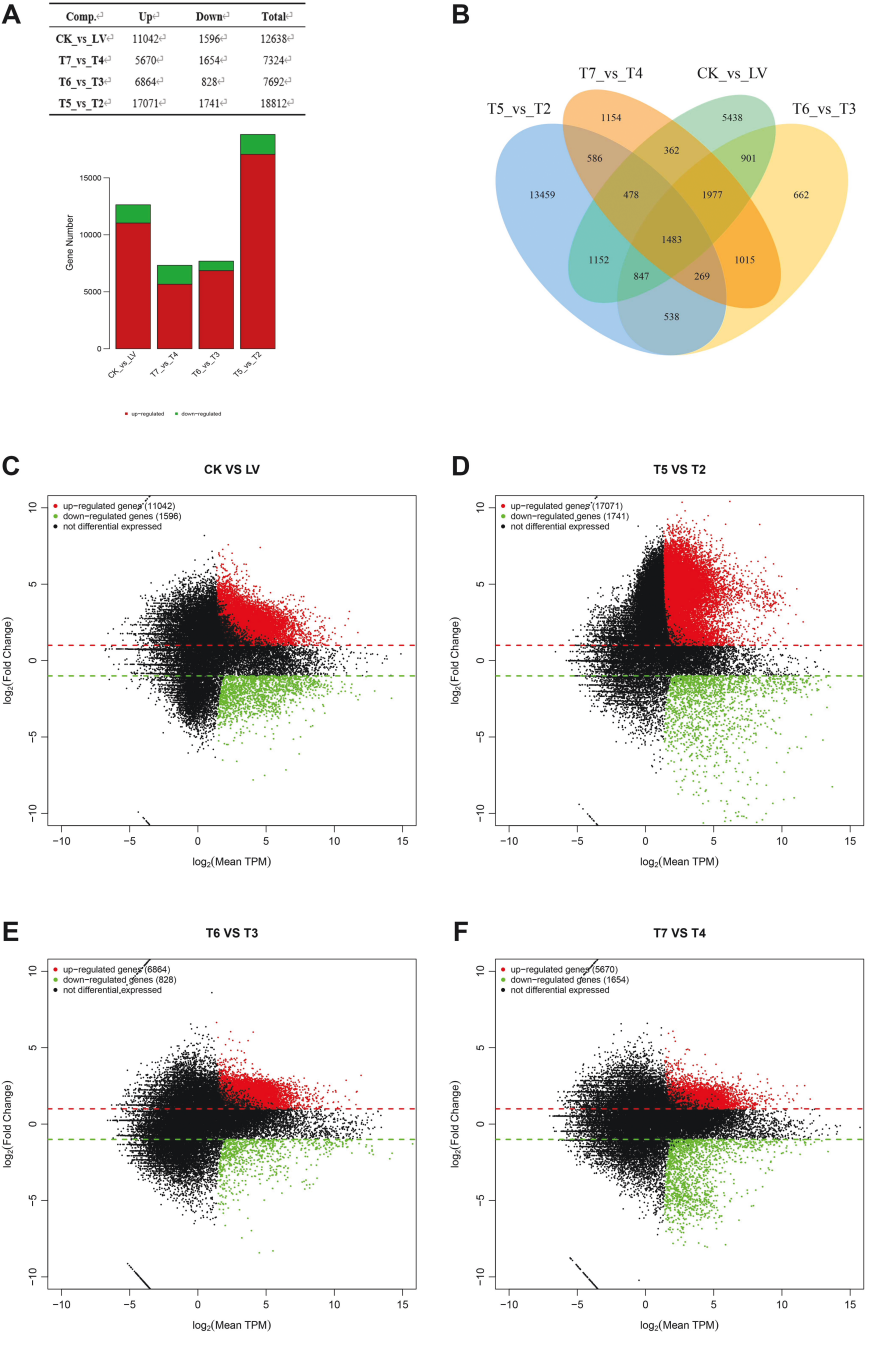
The number and expression patterns of DEGs in different groups. (A) Number and specific dysregulation of DEGs in different groups. (B) Venn plot to summarize DEGs between different paired groups. (C–F) MA plot of DEGs between CK and LV (C), T5 and T2 (D), T6 and T3 (E), and between T7 and T4 (F).

The number of DEGs among different groups after chlorantraniliprole treatment is summarized in Fig. 2A. Detailed DEGs information (i.e., gene ID, log2 Fold Change, q value, etc.) for LV vs. CK, T7 vs. T4, T6 vs. T3, and T5 vs. T2 paired comparisons is shown in Supp Tables S1–S4 (online only), respectively. A total of 1,483 DEGs were observed at each developmental stage after chlorantraniliprole treatment (Fig. 2B). The distribution of upregulated and downregulated genes among different groups was consistent (Fig. 2C–F).

### Functional Enrichment and Annotation of DEGs

**Fig. 3.**
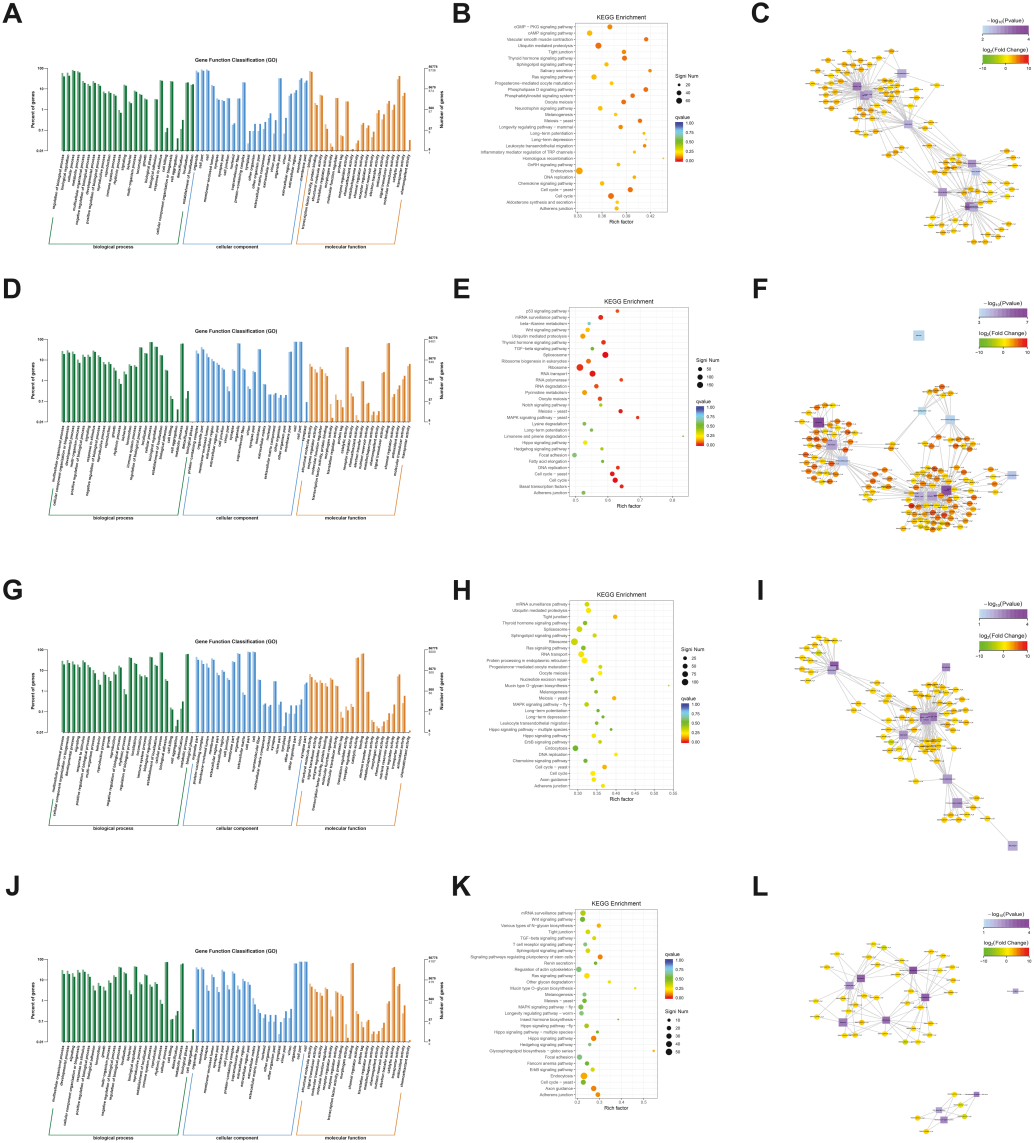
Function enrichment and annotation of DEGs in different paired groups. (A–C) GO annotation (A), KEGG function enrichment (B), and function–gene network (C) of DEGs between CK and LV. (D,F) GO annotation (D), KEGG function enrichment (E), and function–gene network (F) of DEGs between T5 and T2. G-I. GO annotation (G), KEGG function enrichment (H), and function–gene network (I) of DEGs between T6 and T3. (J–L) GO annotation (J), KEGG function enrichment (K), and function–gene network (L) of DEGs between T7 and T4. The light color in GO annotation represents DEGs, while the deep color represents all genes. The size of the points in the KEGG function enrichment represents the number of DEGs, while the color represents the Qvalue. The square nodes in the function–gene network represent the function, while the circle nodes represent correlated genes. The size of the nodes is positively correlated with the degree, and the color represents the pattern of alteration of gene regulation (green: downregulation; red: upregulation).

The GO terms of the DEGs in group LV (compared to CK) were enriched in 26 biological processes, 22 cellular components, and 20 molecular functions (Fig. 3A). The top 30 GO terms were enriched, including tight junctions, cell cycle, and endocytosis (Fig. 3B). Functions with enrichment in the top 10 GO terms were associated with correlated genes and established a function–gene interaction network (Fig. 3C).

GO enrichment of the DEGs in group T2 (compared with T5) revealed 25 biological processes that excluded the biological phase compared with the LV group, 22 cellular components, and 20 molecular functions (Fig. 3D). The top 30 GO functions were enriched, including ribosome, spliceosome, and RNA transport (Fig. 3E). Functional gene networks of DEGs in group T2 were also established (Fig. 3F).

GO enrichment of DEGs in group T3 (compared with T6) revealed 25 biological processes that excluded the biological phase compared with the LV group, 22 cellular components, and 19 molecular functions, excluding chemoattractant activity (Fig. 3G). The top 30 GO functions were enriched, and an interaction network with correlated genes was established (Fig. 3H and I).

As for DEGs in group T4, the GO terms enriched 24 biological processes that excluded biological phase and cell aggregation compared to the LV group, 22 cellular components, and 19 molecular functions excluding chemoattractant activity (Fig. 3J). The functions of the top 30 GO terms were enriched and an interaction network of correlated genes was established (Fig. 3K and L).

### Expression of Detoxification Pathways Correlated with P450 and Glutathione Metabolism

**Fig. 4.**
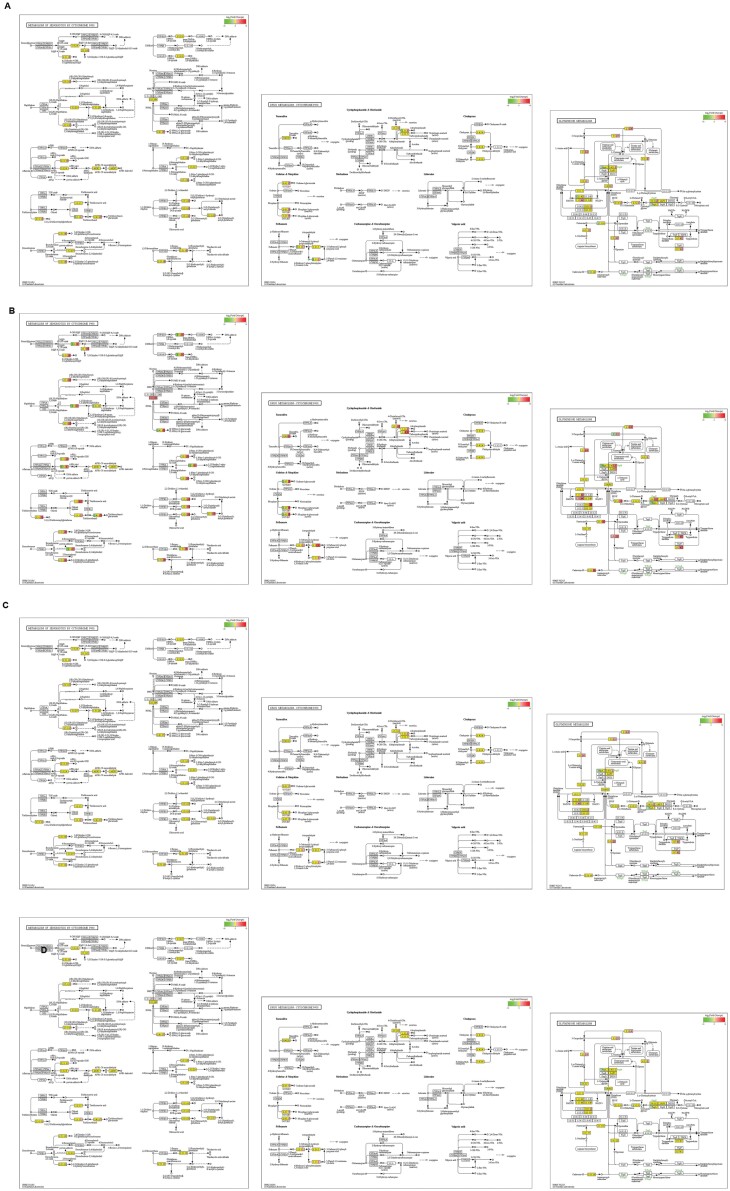
Involvement of DEGs between CK and LV (A), T5 and T2 (B), T6 and T3 (C), and between T7 and T4 (D) in detoxification-related pathways. The order of the pathways is map00980, map00982, and map00480, from left to right. Rectangular nodes represent gene products (such as the enzyme or some RNA regulatory factors), while circular nodes represent compounds (such as substrates or products). The color represents the pattern of alteration of gene regulation (green: downregulation; red: upregulation, yellow: common genes) and the size represents the proportion of the corresponding genes in the products.

According to the KEGG database, two pathways correlated with P450, map00980 (metabolism of xenobiotics by cytochrome P450) and map00982 (drug metabolism-cytochrome P450), and a pathway correlated with glutathione and glutathione metabolism (HYPERLINK ‘https://www.kegg.jp/pathway/map00480+K00799’) were found. A minority of DEGs in different groups were involved in the gene products of P450- and glutathione metabolism-correlated pathways, and the percentage differed among various DEGs (Fig. 4A–D).

### Expression Level of Key Genes

The expression level of TRINITY_DN48242_c7_g4, TRINITY_DN31375_c0_g1, and TRINITY_DN45356_c0_g2 increased significantly after drug treatment, compared to the corresponding level in the LV group. Additionally, the expression level of TRINITY_DN48242_c7_g4 and TRINITY_DN45356_c0_g2 increased significantly after drug treatment in group T7.

## Discussion

Insect sensitivity to insecticides is related to several factors, including biological characteristics and ecology of the species, insecticide safety, and genetic factors (Casida and Durkin 2017, Seesen et al. 2020, Wang et al. 2020). For example, the knockdown mutation of the ryanodine receptor gene inhibits the chlorantraniliprole sensitivity of *Mythimna separata* Walker (Wang et al. 2018). Furthermore, the development of second-generation sequencing has revealed increasing evidence regarding the genetic mechanisms of pesticide resistance in different insect species. Thus, in *Frankliniella occidentalis*, for example, a variety of DEGs identified by transcriptomic analysis and characterization reportedly correlated with different insecticide responses (Gao et al. 2020). Similarly, Gao et al. (2021) reported that the *Plutella xylostella* response to five pesticides was associated with two CYP450 genes, CYP301a1 and CYP9e2, and nine cuticular protein genes (Gao et al. 2018). Additionally, a transcriptomic study demonstrated that pesticide resistance of *Drosophila melanogaster* was likely unrelated to direct metabolic detoxification pathways, but correlated with the restoration of homeostasis. In this study, several DEGs correlated with chlorantraniliprole sensitivity of *T. chilonis* at different developmental stages. The functions of the observed DEGs involved a wide range of signaling pathways, depending on developmental stage. Thus, at the early stages of development, DEG functions mainly involved the spliceosome, RNA transport, and cell cycle, which are critical at that stage (Yi et al. 2018). DEGs are usually upregulated in the early stages. In contrast, at the intermediate and late stages of *T. chilonis* development, functional enrichment revealed that, in addition to the early stage-related functions, DEGs were also involved in endocytosis, and downregulation of DEGs was observed.

Moreover, DEGs were found to be involved in cytochrome P450-related pathways. Cytochrome P450 enzymes (CYP450s) are a superfamily of hemoproteins involved in a variety of reactions, including drug metabolism and xenobiotic degradation (Uehara et al. 2020). CYP450s are reportedly involved in the biotransformation of 80–90% of pharmaceutical drugs and xenobiotics in humans (Pirmohamed and Park 2003), and are also responsible for detoxification of some virulence genes. According to a previous report, silencing of CYP450s in *Spodoptera frugiperda* enhanced the susceptibility of this insect to chlorantraniliprole (Bai-Zhong et al. 2020). Conversely, enhanced chlorantraniliprole resistance by CYP450s was observed in *Chilo suppressalis* Walker, thus demonstrating the involvement of CYP6CV5, CYP9A68, CYP321F3, and CYP324A12 in chlorantraniliprole resistance (Xu et al. 2019). In this study, all DEGs from different developmental stages of *T. chilonis* were observed to mediate two CYP450-correlated pathways, including xenobiotic metabolism and drug metabolism by CYP450, indicating that CYP450s mediate chlorantraniliprole detoxification in *T. chilonis*, and that the inhibition of CYP450-related pathways may induce toxicity in *T. chilonis*. As CYP450 includes numerous isoforms, identifying the specific subtype of CYP450s involved in chlorantraniliprole detoxification would benefit the control effects of *T. chilonis* (Waring 2020).

Glutathione also plays a critical role in immune system function and has been demonstrated to possess antioxidant and detoxifying activities by binding to drugs or toxins through sulfhydryl (Dröge and Breitkreutz 2000, Wu et al. 2004, Forman et al. 2009). Glutathione-related indicators, such as glutathione-S-transferase and total glutathione, are considered as biomarkers correlated with critical insect physiological functions; furthermore, glutathione-related indicators reportedly participate in chlorantraniliprole resistance. For instance, Yin et al. (2021) focused on chlorantraniliprole resistance of *Plutella xylostella* and found that glutathione S-transferase played a vital role. Here, we found that the GSH metabolism pathway was related to DEGs identified from different developmental stages of *T. chilonis*, indicating that GSH metabolism might be another mechanism underlying chlorantraniliprole detoxification in *T. chilonis*.

Our results demonstrate that DEGs which correlated with chlorantraniliprole sensitivity in *T. chilonis* were distinct and had various functional roles at different developmental stages. Chlorantraniliprole detoxification in *T. chilonis* was associated with CYP450- and glutathione-related pathways. These findings provide novel insights for balancing chemical and biological pest control methods for a more sustainable and highly productive agriculture.

## Supplementary Material

ieac044_suppl_Supplementary_Table_S1

ieac044_suppl_Supplementary_Table_S2

ieac044_suppl_Supplementary_Table_S3

ieac044_suppl_Supplementary_Table_S4
